# Editorial

**Published:** 1916-12

**Authors:** 


### EDITORIAL

The Atlanta Journal-Record of Medicine has its Business Office at 23 West Alabama Street where all business communications should be sent.

Remittances are payable to The Journal-Record of Medicine.

Reprints of Original Articles will be furnished at cost price. Requests for them had best be made on the Manuscript.

Upon request, we will present, postpaid, to each contributor of an original article, twenty copies of The Journal-Record of Medicine containing such article.

### THE FULTON COUNTY MEDICAL SOCIETY

In making a new start for the year 1917 a few reflections concerning the Fulton County Medical Society may not be amiss.

Situated in the county containing the largest city in the state, and in numbers outclassing any of the other county societies, certain responsibilities naturally devolve upon lt. This society is expected to be progressive, to set an enegertic pace, and to hold such interesting meetings that medical visitors to Atlanta (and they are many) may find then both interesting and instructive. This Society should be and can be a clinical "clearing house", where cases of interest can be exhibited and discussed, where obscure manifestations of disease or bizarre expressions of pathologic states may receive the focus of minds trained and matured by experience. Thus, in a medical way, its sessions may constitute a "feast of of reason and flow of the soul."

One most helpful point in the promotion of "ginger" in these meetings is the avoidance of tedious and lengthy discussions. The rule limiting general discussions of papers or cases to five minutes for each individual is a wise one. The pith of nealry every viewpoint can certainly be expressed in five minuttes, and when the remarks are strung out indefinitely they are liable to constitute what Shakespeare calls "admnable itersion."

The following lines by William Cowper, though not intended to describe a discussion in medical societies will apply quite fitly:—

"A tale should be judicious, clear, succinct;
The language plain, and incidents well linked;
Tell not as new what everybody knows,
And, new or old, still hasten to a close;
There centering in a focus round and neat,
Let all your rays of information meet."

The new officers for the present year are President, Dr. R. B. Ridley, Jr., an earnest and capable man, imbued with a sincere desire for the Society's welfare; Vice President, Dr. H. M. Lokey, an enegertic and popular member, one in whose veins flows more than the ordinary amount of the milk of human kindness; and Secretary, Dr. Walpole Brewer, experienced in the work, and who is more a man of deeds than of words.

May the members pull together for the common good and may this year so fraught with distress for some of the nations of the world, but so laden with manifold blessings for our own beloved country, be a banner year for the FULTON COUNTY MEDICAL SOCIETY.

